# Familial Lung Cancer: A Brief History from the Earliest Work to the Most Recent Studies

**DOI:** 10.3390/genes8010036

**Published:** 2017-01-17

**Authors:** Anthony M. Musolf, Claire L. Simpson, Mariza de Andrade, Diptasri Mandal, Colette Gaba, Ping Yang, Yafang Li, Ming You, Elena Y. Kupert, Marshall W. Anderson, Ann G. Schwartz, Susan M. Pinney, Christopher I. Amos, Joan E. Bailey-Wilson

**Affiliations:** 1 National Human Genome Research Institute, National Institutes of Health, Baltimore, MD 21224, USA; musolfam@mail.nih.gov (A.M.M.); clairelsimpson@uthsc.edu (C.L.S.); 2 Department of Genetics, Genomics and Informatics, University of Tennessee Health Science Center, Memphis, TN 38103, USA; 3 Mayo Clinic, Rochester, MN 55904, USA; mandrade@mayo.edu (M.d.A.); yang.ping@mayo.edu (P.Y.); 4 Department of Genetics, Louisiana State University Health Sciences Center, New Orleans, LA 70112, USA; dmanda@lsuhsc.edu; 5 Department of Medicine, University of Toledo Dana Cancer Center, Toledo, OH 43604, USA; colette.gaba@utoledo.edu; 6 Geisel School of Medicine, Dartmouth College, Lebanon, NH 03766, USA; yafang.li@dartmouth.edu (Y.L.); christopher.i.amos@dartmouth.edu (C.I.A.); 7 Cancer Center, Medical College of Wisconsin, Milwaukee, WI 53202, USA; myou@mcw.edu (M.Y.); elenakupert@gmail.com (E.Y.K.); andermw@ucmail.uc.edu (M.W.A.); 8 Karmanos Cancer Institute, Wayne State University, Detroit, MI 48226, USA; schwarta@karmanos.org; 9 Department of Environmental Health, University of Cincinnati College of Medicine, Cincinnati, OH 45202, USA; pinneysm@ucmail.uc.edu

**Keywords:** lung cancer, susceptibility, major gene, linkage, family studies

## Abstract

Lung cancer is the deadliest cancer in the United States, killing roughly one of four cancer patients in 2016. While it is well-established that lung cancer is caused primarily by environmental effects (particularly tobacco smoking), there is evidence for genetic susceptibility. Lung cancer has been shown to aggregate in families, and segregation analyses have hypothesized a major susceptibility locus for the disease. Genetic association studies have provided strong evidence for common risk variants of small-to-moderate effect. Rare and highly penetrant alleles have been identified by linkage studies, including on 6q23–25. Though not common, some germline mutations have also been identified via sequencing studies. Ongoing genomics studies aim to identify additional high penetrance germline susceptibility alleles for this deadly disease.

## 1. Overview

Lung cancer is the most deadly of all cancers, and has been the leading cancer killer in men since the early 1950s and of women since 1987. The mortality rate for this disease was the greatest of any cancer from 1950–1988, primarily due to the increases in tobacco smoking habits [1]. While lung cancer incidence and mortality have decreased, lung cancer still kills more people than any other cancer in the United States. An estimated 158,080 Americans will die of lung cancer in 2016, corresponding to roughly one in four cancer deaths [2]. Part of the reason for this large death toll is that the disease is often detected at advanced stages of malignancy (stage 3 or 4), at which point treatment is far less effective [3].

Indeed, early detection of lung cancer can be pivotal in saving lives. Recent evidence from the National Lung Screening Trial (NLST) has shown that low-dose computed tomography has been effective at reducing lung cancer mortality by 20% [4]. The American Cancer Society now recommends that clinicians should discuss screening with healthy patients over the age of 55 with a history of smoking [5]. Identification of individuals at particularly high risk of lung cancer due to inherited (germline) susceptibility alleles would contribute to early detection and possibly to prevention through targeted anti-smoking efforts.

Lung cancer treatment has improved steadily over the past decade. Oral targeted therapies such as osimertinib [6] target oncogenic mutations in genes such as *EGFR*, *ALK*, *ROS1* and *RET* [7,8,9,10,11]. These oral targeted therapies have been successful in improving clinical outcomes. Drug resistance can be a problem; some tumor cells mutate to a resistant state and predominate after vulnerable cells are killed off, while others activate alternate pathways that can inhibit the treatment [9]. There are promising efforts to combat this problem [9,12,13,14]. Most recently, immune checkpoint inhibitors have transformed the treatment of lung cancer, and appear to offer the promise of achieving durable remissions in some patients [15,16,17,18,19,20]. Treatment of lung cancer is changing rapidly, and a detailed review is beyond the scope of this article. However, to aid in the development of new drugs and preventative measures, it is necessary to understand the genetics that underpin the development of lung cancer tumors. Since cancers arise through an accumulation of deleterious somatic mutations, environmental or biological risk factors that favor this process (including inherited deleterious germline genetic variants) are important.

## 2. Environmental Effects

Lung cancer is often cited as an example of a malignancy that is almost exclusively determined by environmental factors. In addition to the well-known risks associated with tobacco smoking [21,22,23,24], occupational hazards from mining, asbestos exposure, shipbuilding, and petroleum refining are documented causes of the disease [25]. However, not all environmental effects are deleterious; dietary studies have shown that a diet high in carotene containing fruits and vegetables reduce risk; the carotenoids α-carotene and β-cryptoxanthin have been shown to significantly decrease lung cancer risk [26], although other studies have shown this relationship to be inconclusive or not statistically significant [27,28].

## 3. Inhalation of Tobacco Smoke

Of all the environmental factors that affect lung cancer, the smoking of tobacco products has the greatest impact. It is directly responsible for approximately 85% of lung cancer risk [29,30,31,32].

The effects of tobacco smoke inhalation are well documented. Incidence of lung cancer is correlated with the cumulative amount and the duration of tobacco products smoked in a dose–response relationship [22]. Smoking cessation results in a decreased risk of the disease, with the decrease being related to the time elapsed since the individual stopped smoking [22,29]. It has also been found that lung cancer rates and smoking rates differ by geographical regions [33] and between males and females. About 90% of lung cancers in men and women were directly attributable to tobacco smoking [34,35].

Tobacco smoke has been found to cause both mutations and loss of heterozygosity at oncogenes and tumor suppressor genes known to be causal in lung carcinogenesis. Kondo et al. have shown a significant (*p* < 0.001) dose-response relationship between the number of cigarettes smoked and *TP53* mutation frequency in lung cancer patients [36]. Mutations in *KRAS* and several genes on 15q.25 have also been found to be significantly associated with smoking in multiple populations [37,38,39,40,41,42,43]. These results suggest that somatic mutations in these genes may be due to carcinogens or mutagens found in tobacco smoke.

## 4. Nonsmoker Lung Cancer Risk

While personal tobacco smoking can explain the vast majority of lung cancer cases, this is not the sole cause. An estimated 10%–25% of lung cancer cases occur in nonsmokers [44]. Environmental tobacco smoke (ETS)—also called passive smoking or secondhand smoking—is a factor in at least some of these cases. ETS has been shown to increase the risk of developing lung cancer in nonsmokers by at least 20% [25]. A meta-analysis of 22 independent studies showed that workplace ETS exposure increased lung cancer risk by 24%, and showed high positive correlation with exposure duration [45].

However, ETS is only thought to be responsible for 16%–24% of lung cancer in nonsmokers, and even as more stringent laws against tobacco smoking in public are passed, the number of lung cancer cases in nonsmokers may actually be increasing [46]. Many studies have shown that somatic mutations are more common at certain genetic loci, and thus these genes are thought to be important lung cancer driver genes. For instance, somatic mutations in *EGFR* and translocation of *ALK* (both genes known to affect lung cancers) occur at higher rates in nonsmoking lung cancer patients than in the tumors of smoking lung cancer patients [39,40,47].

## 5. Lung Cancer Familial Clustering

Epidemiological evidence has suggested that lung cancer may show familial aggregation after proper adjustments for tobacco smoking and other environmental factors. This means that differential susceptibility to lung cancer may be inherited in an oligogenic fashion.

The first major evidence for the familial aggregation of lung cancer was published 50 years ago by Tokuhata and Lilienfeld [48,49], which found that lung cancer mortality increased in smoking relatives of cases as compared to smoking relatives of controls. More interestingly, they also found that nonsmoking relatives of cases had a higher risk of lung cancer than nonsmoking relatives of controls. For the first time, this suggested that a genetic component was involved in lung cancer risk.

After this first evidence, other studies began to report familial aggregation of lung cancer. A case-control study in southern Louisiana showed a 2.4-fold higher risk for individuals with a familial relationship to a lung cancer proband after controlling for environmental factors such as tobacco smoking [50]. Further studies involving patients from Saskatchewan [51], Utah [52,53], Houston [54], and Detroit [55] demonstrated a higher risk of lung cancer in individuals with an affected family member after adjusting for smoking histories. The Detroit study showed this risk was higher in African-American families [55], while the Utah study observed higher relative risks for female relatives of female probands vs. male relatives of male probands [53]. The largest familial aggregation study came from Iceland, and obtained familial risks of 2.69 for parents of cases compared to controls and 2.02 for siblings [56]. A meta-analysis performed on 28 case-control studies and 17 cohort studies reported an approximately two-fold increase associated with family history of lung cancer [57]. A more recent analysis conducted by the International Lung Cancer Consortium (ILCCO) in 2012 with 24,000 cases and 23,000 controls reported a significant 1.5-fold increase in risk due to family history after adjustment for environmental confounders [58]. This study also confirmed a higher risk in African-Americans (risk of 2.09) than Caucasians (risk of 1.53).

Studies involving never-smoking lung cancer cases are rare, though Schwartz et al. [59] showed that lung cancer risk was increased in younger nonsmoking relatives of cases compared to similarly aged relatives of controls. Mayne et al. [60] reported family history of lung cancer was associated with increased risk in a study involving nonsmokers and former smokers after adjusting for age and smoking status. The 2012 ILCCO study also found a significant 1.25-fold risk increase for family history in nonsmokers [58].

However, it is well known that familial aggregation of a disease or trait does not prove inheritance of genetic risk variants, since clustering of exposure to environmental risk factors within close relatives is also a possible cause of such familial aggregation of cancer. After observing significant familial aggregation, segregation analyses are a classic method of determining whether genetic models that include risk due to a major gene (i.e., high penetrance genetic variants) are consistent with the pattern of disease in multiplex families. Rejection of purely environmental models and good fit of models that include a genetic component adds evidence that familial aggregation is at least partially due to genetic variants.

## 6. Lung Cancer Segregation Analyses

It is clear that lung cancer displays familial aggregation in affected families, even after adjusting for environmental factors such as tobacco smoking. The logical next step is to determine if transmission patterns within families are representative of a major high penetrance risk locus. Genetic segregation analyses of the southern Louisiana study [50] mentioned above [61,62,63,64] found that the data were compatible with a Mendelian codominant inheritance of a rare major autosomal gene acting in conjunction with smoking and polygenic risk factors that produces an earlier age of lung cancer onset. These families were collected via a population-based study design under single ascertainment. All segregation analyses corrected for this single-ascertainment. Further analyses of these data using a Gibbs sampler method (modeling environmental interactions) showed evidence of a major dominant susceptibility locus that acts in conjunction with tobacco smoking [65]. Both the dominant and codominant models predicted a rare disease allele frequency with a very small number of homozygous allele carriers. Further studies with nonsmoking lung cancer probands from Detroit [66] and Taiwan [67] found strong evidence for the codominant Mendelian model with modifying environmental effects.

The Louisiana, Detroit, and Taiwan studies demonstrate statistical evidence for at least one major gene that acts in tandem with tobacco smoking to increase lung cancer risk. Segregation analyses are not sufficient on their own to prove the existence of a major susceptibility locus, because not all possible models can be tested. The segregation analyses performed for lung cancer suggested the joint action of major risk alleles, polygenic risk factors, and environmental risk factors in the etiology of this disease. As described above, epidemiologic studies have delineated the environmental risk factors. To further understand the genetic components of risk, genome-wide association studies and linkage studies have been useful.

## 7. Lung Cancer Genome-Wide Association Studies

Genome-wide association studies (GWAS) are designed to identify common low penetrance genetic risk alleles with moderate effects on lung cancer risk. GWAS use a large sample of unrelated individuals and 300,000 or more markers (usually single nucleotide polymorphisms, or SNPs) that are spread across the genome. One then attempts to find associations between these markers and the given phenotype. Significant association implies that a risk variant (allele) at this location or in linkage disequilibrium with this marker increases risk of the disease. Several recent GWAS have provided highly significant and reproducible results for lung cancer.

A region on chromosome 15q was initially identified as significantly associated with lung cancer risk by three different studies of European ancestry samples with approximately 5000 cases each [41,42,43]. This region contains the neuronal acetylcholine receptor gene cluster subunits *CHRNA3, CHRNA5*, and *CHRNB4*. All three studies identified the 15q25 region as associated with an increased risk of lung cancer—with relative risks of 1.29 for heterozygotes and 1.80 for homozygotes. There was some dissent between the three studies as to whether the region was directly associated with lung cancer or indirectly associated through smoking. Thorgeirsson et al. [43] suggested the region affected smoking behavior, Amos et al. [41] found stronger effects on lung cancer that remained highly significant after smoking behavior adjustments, while Hung et al. [42] found no association with smoking.

Other GWAS performed with European ancestry individuals have found associations with 6p21 and 5p15 [41,42,68,69]. An additional meta-analysis with approximately 900 cases and 30,000 controls confirmed this association in Europeans at 5p and 6p21, as well as 15q25 [70]. Further studies have confirmed the 15q25 results in Europeans [71] and African-Americans [72].

Replication has also been achieved in several Asian populations. Chinese GWAS with approximately 2000 cases and 3000 controls have identified significant SNPs associated with lung cancer (*p*-value < 10^−8^). Three of the SNPs were located at well-known loci (5p15 and 3q28), and three were located in loci novel in Chinese populations [73]. A follow-up study with approximately 7500 cases and 7500 controls found additional susceptibility loci at 10p14, 5q32, and 20q13, with each locus having a single genome-wide significant SNP [74]. The SNP at 5q32 was also shown to be significantly associated with smoking dosage (odds ratio of 1.09).

A Japanese study with approximately 6000 cases and 13,000 controls replicated the 5p15, 6p21, and 3q28, with odds ratios (OR) of 1.41, 1.18, and 1.25, respectively [75]. A novel locus was also identified at 17q24 (OR = 1.20). Current GWAS results have focused on increasing predictive value, as GWAS usually identify variants with low penetrance. Predictive value can be increased by considering these variants for their cumulative effect in biological pathways, which has been shown to increase the OR of developing lung cancer by 4.68 [76]. Other studies have combined lung cancer GWAS with data from other cancer GWAS to find shared pathways. One study uses a combination of lung cancer, prostate cancer, and breast cancer to determine the pathways associated with significantly associated genes, and found that genes in the nerve growth factor, epidermal growth factor, and fibroblast growth factor pathways were present in all three cancers [77]. The study also found that lung cancer on its own is significantly associated with immune related and cell organization pathways.

## 8. Lung Cancer Linkage Analyses

While GWAS are most highly powered to identify common risk alleles which tend to be of low penetrance (polygenic component of risk), linkage analyses are designed to find rare high penetrance genes with large effects. Linkage analysis uses family pedigrees in an attempt to find co-segregation of alleles through multiple generations at a genetic susceptibility locus (e.g., the lung cancer phenotype risk locus) and a known genetic marker (commonly a polymorphism like a SNP or microsatellite). Linkage analysis is very powerful at detecting loci that are highly penetrant (after adjustments for environmental factors). Power decreases as the allele becomes more common and less penetrant. Specifically with respect to lung cancer, tobacco smoking is such a significant risk factor that it must be controlled in any linkage analysis. Once properly accounted for, the power to detect linkage with a major gene will increase.

The first evidence for linkage of a lung cancer susceptibility locus was published by Bailey-Wilson et al. [78] in 2004. The putative locus was localized to a region on 6q23–25. Patients were identified from eight sites across the United States by the Genetic Epidemiology of Lung Cancer Consortium (GELCC). For each family member, data on cancer status, birthdates, age at diagnosis, and vital statistics were collected, as well as archival tissue and blood/saliva. Cancer status was verified by medical records, pathology reports, cancer registry records, and death certificates for the majority of all lung cancer patients (69%). The remainder was confirmed through the reporting of multiple family members.

Fifty-two initial families were selected for genotyping, which consisted of 392 microsatellite markers. Analysis was performed using both parametric and nonparametric linkage methods. The parametric analyses assumed a dominant model with 10% penetrance in carriers and 1% in non-carriers. This model weighted information from affected individuals only. The model was chosen due to the high amount of uncertainty about the strength of the relationship between smoking and lung cancer risk, and the fact that no software currently exists to perform multipoint linkage analyses while modeling complex gene–environment interactions. Further, 90% of the affected individuals were known to smoke, so weighting affected individuals in this dominant, low penetrance model also jointly allowed for smoking status. The primary two-point analyses were performed by FASTLINK, and multipoint analyses were performed by SimWalk2. Additional, more complex two-point analyses that took age and pack-years of cigarette smoking using the genetic regressive model based on the results found by Sellers et al. [61] were performed; nonparametric analyses were performed using SOLAR (binary phenotype) and mixed effects Cox regression models (quantitative phenotype).

Linkage analyses are typically quantified using the LOD score, which is a statistical measure of the strength of the evidence in favor of linkage between a given genetic variant and the phenotype being studied. LOD scores are cumulative and can be added across multiple families to obtain an overall LOD score for the entire data set. A LOD score equal to or above 3.3 or higher corresponds to a *p*-value of 4.9 × 10^−5^, and is considered genome-wide significant [79]. The LOD score is appropriate only when all families in the sample are affected by the same causal gene. However, it is known that many complex diseases have substantial locus heterogeneity (i.e., different families have causal variants in different genes). For example, *BRCA1* and *BRCA2* both harbor high-risk genetic variants that cause increased risk of breast cancer in different families. In the presence of substantial locus heterogeneity, a heterogeneity LOD score (HLOD) which estimates the proportions of families linked to a particular locus is the more appropriate test [80]. HLOD scores use the same level of genome-wide significance (≥3.3) as LOD scores.

Multipoint analyses yielded a maximum heterogeneity LOD (HLOD) score of 2.79 at 155 cM on chromosome 6q23–25 in all families, a score slightly below genome-wide significance. Further analyses using families with at least four affected individuals or at least five affected individuals yielded the genome-wide significant HLOD scores of 3.47 and 4.26, respectively. The two-point analyses and the nonparametric analyses both supported linkage to this region. This was the first time that any genome-wide significant linkage to familial lung cancer had been found. This implies that some rare genetic variants exist that act to substantially increase risk of lung cancer.

Additional genotyping was conducted on 93 high risk lung cancer families in an update to the initial GELCC study in 2010 [81]. Approximately 400 markers were genotyped, and the same dominant low penetrance model was used. Again, this analysis revealed a region on 6q with a maximum HLOD score of 4.67 in families with at least five affected individuals. This new study also revealed that lung cancer risk was higher among individuals who were carriers of the linked disease haplotype than non-carriers of the linked haplotype, even if the carrier was a never-smoker. Further, additional evidence for suggestive linkage was found on chromosomes 1q, 5q, 8q, 9p, 12q, 14q, and 16q.

## 9. Lung Cancer Risk and Germline Variants

Some causal germline variants have been identified in a small proportion of familial lung cancer pedigrees. Mutations in *TP53* have long been known to cause Li-Fraumeni syndrome [82], which causes an increased risk of lung cancer, among other cancers. More recently, a de novo mutation in a tumor of a Li-Fraumeni patient was found to have a deleterious mutation in *CHEK2* [83]. Patients with DICER1 syndrome—which also causes an increase in lung cancer risk—have been found to have germline mutations in the ribonuclease *Dicer 1* gene [84]. Another germline mutation in *SFTPA1* was identified by Sanger sequencing in a family that suffered from both familial idiopathic pneumonia and lung cancer [85].

Sanger sequencing of some lung cancer families found an association between lung cancer and the T790M mutation in *EGFR* [86,87,88,89]. The T790M mutation in *EGFR* has also been reported to disproportionately affect risk of lung cancer in never-smokers as opposed to heavy smokers, with the nonsmoking group having approximately a 31% risk increase [90]. This study was based on a small sample of individuals, and more work is needed to confirm these estimates. Whole exome sequencing (WES) was recently used to identify a novel germline mutation in *HER2* in a Japanese family with a history of lung cancer [91]. Direct sequencing of a germline BAP1 protein detected a truncation in 53 individuals from families with a high risk of hereditary cancers, including lung cancer [92]. The GELCC has also used WES and Sanger sequencing to propose that germline mutations in the tumor suppressor *PARK2* and the G-protein regulator *RGS17* genes result in increased lung cancer susceptibility in a small number of families [93,94]. The causal germline susceptibility genes have not yet been identified for the majority of high-risk lung cancer families.

## 10. Conclusions and Prospects for the Future

In this paper, we have provided a brief review of the history of familial lung cancer. We long knew that lung cancer risk was increased by environmental factors such as tobacco smoking, but described how familial clustering studies published in the 1960s gave the first hints that lung cancer also had a genetic component. Other clustering studies followed in suit, and soon it was well-established that having a family history of lung cancer increased one’s risk of developing the disease. We then described segregation analyses that were performed, and using the family data determined that—in addition to risk due to smoking—lung cancer risk may in part be caused by a major autosomal dominant susceptibility locus plus a polygenic component; the finding that a major gene may be acting in tandem with tobacco smoking was confirmed by others. This allowed both GWAS and linkage studies on lung cancer to be performed to find these risk variants. GWAS have located multiple associations between lung cancer risk and genetic loci. Some of the more prominent associations that have been replicated are found on 15q25, 6p21, and 5p15. Many of these findings have also been replicated in multiple ancestry groups. Linkage studies have tended to be much rarer than GWAS, because they require costlier and more time-consuming efforts to collect the necessary family data. Nonetheless, linkage studies have identified significant signals on 6q23–25, and suggestive signals on multiple chromosomes. Several other rare genetic variants—such as in the *EGFR* and *TP53* genes—have been identified that appear to increase lung cancer risk in a small number of families.

While our understanding of familial lung cancer has come a long way, there is still much more to be done. One of the major issues hindering linkage studies is that there are few powerful and reliable ways to include covariates (such as tobacco smoking information) in linkage analysis. Important environmental effects can be controlled (e.g., focusing on performing affected-only analyses to account for the uncertainty between lung cancer risk and the environmental effect), but the ability to correctly model these relationships in linkage studies has remained elusive. The existing methods that attempt this all have some shortcomings. The rise of population-based GWAS and the supposed “death” of family-based linkage studies in the early to mid-2000s led to the stagnation of linkage software development, including work on the addition of covariates to the analysis. As the value of family-based studies reemerged in recent years, hopefully this will spur the advancement of more linkage software that includes covariates into its statistical framework. Hopefully, other factors such as gene–environment and gene–gene interactions can also be included into this framework.

GELCC sequencing studies are ongoing in our previously collected GELCC familial lung cancer pedigrees. To further localize the causal variant on 6q23–25, targeted sequencing of the region is being analyzed in our nine most highly-linked families. Whole exome sequencing (WES) on 25 of the most informative families from our previous studies is also ongoing.

The GELCC is still actively seeking out families with a strong history of lung cancer, with new participant families being collected and genotyped. Since the 2010 study, we have acquired an additional 25 extended families with a history of lung cancer and adequate biospecimens for family studies. We have also obtained biospecimens on over 1000 familial lung cancer cases as well as controls who are frequency matched for age, smoking status, and ethnicity. A familial case–control GWAS is underway using these samples. In addition, our new highly-aggregated lung cancer families have been genotyped, and linkage analyses on these newest families are currently underway. These familial lung cancer analyses aim to allow us to identify novel lung cancer risk loci. In addition, the linkage study design of the new families has been chosen since lung cancer has a very high phenocopy rate, with some familial aggregation seen by chance. This allows for selection of only families that are segregating a linked haplotype for future whole exome or whole genome sequencing and other genomics studies, thus increasing power to detect germline risk variants. There are other ongoing studies as well. The majority of these are population-based studies, such as a study in China that is recruiting women from the Xuan Wei County [95]. These women are nonsmokers, yet have the highest rate of lung cancer in China, leading researchers to hypothesize a significant genetic component that has yet to be identified. Ongoing studies recruiting families is rarer, but the INHERIT EGFR study is looking for patients with *EGFR* mutations in their lung cancer tumors to determine if these mutations are somatic or were inherited in the germline [95]. Other family-based studies are looking specifically at lung cancer risk in African-American families and the relationship between smoking cessation and lung cancer.

There have been great strides made in the understanding of familial lung cancer. We know that lung cancer aggregates in families and mortality increases in individuals with a relative affected with lung cancer. Segregation analyses suggested that at least one major gene affects lung cancer and provided a working model for parametric linkage analyses. Genome-wide association studies have identified common low penetrance variants conferring moderate risk—particularly in the 15q.25, 5p15, and 3q28 regions. As is common in other hereditary cancers, locus heterogeneity for high penetrance germline risk variants is observed in familial lung cancer. Linkage analyses have identified a high penetrance risk region to 6q23–25, and targeted sequencing analysis is currently underway to identify the causal variant. Germline mutations in multiple genes—particularly in *EGFR*—have been reported that increase risk of lung cancer in small numbers of families exhibiting various rare cancer syndromes. However, the genetic basis of risk in most families with a strong history of lung cancer has not yet been elucidated, and many genomic studies are ongoing in an attempt to remedy this.
